# Whole-Genome Sequence of Salmonella enterica subsp. enterica Serovar Typhimurium Strain WG49 and Escherichia coli Strain WG5 Used in South Africa for Phage Detection in Water Samples

**DOI:** 10.1128/genomeA.00372-18

**Published:** 2018-06-28

**Authors:** L. Bothma, D. Gonzalez-Ibeas, C. Mienie, C. C. Bezuidenhout, R. Adeleke

**Affiliations:** a Unit for Environmental Sciences and Management, North-West University, Potchefstroom, South Africa; b Agricultural Research Council, Institute for Soil, Climate and Water, Pretoria, South Africa

## Abstract

Salmonella enterica subsp. *enterica* serovar Typhimurium WG49 is widely used for enumeration of F-specific RNA (F-RNA) coliphages in water. Escherichia coli WG5 is broadly used for the detection and enumeration of somatic coliphages in water samples. We report here the genome sequences of these bacterial strains used in South Africa under ISO methods 10705-1 and 10705-2.

## Abstract (precis)

Salmonella enterica subsp. *enterica* serovar Typhimurium WG49 is widely used for enumeration of F-specific RNA (F-RNA) coliphages in water.

## GENOME ANNOUNCEMENT

Bacteriophages are biological indicators of fecal contamination of water acting as surrogates for enteric viruses harmful to humans (1–3). Coliphages are phages that infect Escherichia coli. Different E. coli strains have been used as phage hosts in laboratory assays. Some researchers advocate the use of a specific local strain for each country. However, E. coli strain WG5 is broadly used under standardized protocols (ISO method 10705-2 [4]) and is available in the ATCC repository (ATCC 700078). F-Specific RNA (F-RNA) phages are similar to other pathogenic human enteric viruses (e.g., hepatitis A) (3, 5). A method for the enumeration of F-RNA phages was developed by transforming a Salmonella strain with a K-12 plasmid coding for the F-pili of E. coli (6). Following this approach, Salmonella Typhimurium WG49 is broadly used under standardized protocols (ISO method 10705-1 [7]) and is also available in the ATCC repository (ATCC 700730). These strains are used in South Africa for the analysis of water quality in several regions (5, 8, 9) as a reliable and economically affordable method.

Bacterial DNA was isolated with a NucleoSpin tissue kit (Macherey-Nagel) and subjected to Nextera XT DNA library preparation according to Illumina (USA) instructions. Normalized libraries were run on an Illumina MiSeq sequencer (2 × 300-nucleotide [nt] paired-end reads, 1,570,000 reads, 100× estimated coverage). Read quality was evaluated with FastQC (https://www.bioinformatics.babraham.ac.uk/projects/fastqc/). Reads of quality lower than 35 and lengths less than 45 nt were filtered with Sickle (10). Genome assembly was carried out with SPAdes (11) with default parameters. The assembly was evaluated with QUAST (12).

The E. coli WG5 strain has a genome length of 4,513,988 nt, assembled in 151 scaffolds longer than 500 nt and with an *N*_50_ value of 129,141 nt. Annotation was performed with Rapid Annotation using Subsystems Technology (RAST) (13), giving rise to 4,345 protein coding regions, 31 rRNAs, and 83 tRNAs. Only one region was identified as an incomplete prophage by PHAST (14). Two high-quality and nine questionable clustered regularly interspaced short palindromic repeat (CRISPR) sequences were identified by CRISPRFinder (15). Out of the 20 spacers derived from them, only 1 was found to target the known Salmonella phage SJ46. No antibiotic resistance genes were identified by ResFinder (16), but 15 were found by the Antibiotic Resistance Genes Database (ARDB) finder (17).

The Salmonella Typhimurium WG49 strain has a 4,868,868-nt length, assembled in 53 scaffolds longer than 500 nt, with an *N*_50_ value of 299,825 nt. Annotation was performed with RAST (13), giving rise to 4,788 protein coding regions, 31 rRNAs, and 83 tRNAs. Seven regions were identified as potential prophages by PHAST (14), four of them being flagged as “intact,” two as “incomplete,” and one as “questionable.” Three confirmed and two questionable CRISPR sequences were identified by CRISPRFinder (15). Out of the 46 spacers derived from them, similar sequences were found on bacterial genomes falling on annotated CRISPR regions, but none were found to target known sequenced phage genomes. No antibiotic resistance genes were identified by ResFinder (16), but 15 were found by the Antibiotic Resistance Genes Database (ARDB) finder (17).

### Accession number(s).

The whole-genome shotgun sequencing is described in the GenBank BioProject no. PRJNA434049. Raw reads are available from the Sequence Read Archive (SRA) (accession no. SRR6804879 for Salmonella Typhimurium WG49 and SRR6804878 for E. coli WG5). The genome sequences have been deposited at DDBJ/ENA/GenBank under accession no. PXZB00000000 for Salmonella Typhimurium WG49 and PYBI00000000 for E. coli WG5. The versions described in this paper are PXZB01000000 for Salmonella Typhimurium WG49 and PYBI01000000 for E. coli WG5.
